# The importance of drug checking outside the context of nightlife in Slovenia

**DOI:** 10.1186/s12954-018-0208-z

**Published:** 2018-01-12

**Authors:** Matej Sande, Simona Šabić

**Affiliations:** Association for Drug Related Harm Reduction DrogArt, Prečna ulica 6, 1000 Ljubljana, Slovenia

**Keywords:** Drug checking, Harm reduction, Injecting drug users, Early Warning System, Evaluation, NPS, Drug adulterants

## Abstract

**Background:**

The main purpose of the research was to evaluate the implementation of the drug checking service in Slovenia and to obtain the opinion of users included in harm reduction programmes for high-risk drug users and of drug users in nightlife settings on drug checking, the reasons for drug checking, and their attitude towards adulterants in the drugs that they use.

**Methods:**

The two final unrepresentative research samples included 102 respondents from harm reduction programmes and 554 respondents from the online sample. The questionnaire was designed based on analysis of the interviews conducted with professionals from the programmes, who took part in the drug checking project, and based on previous research on drug use in nightlife.

**Results:**

The main findings related to users’ opinions on the drug checking service are that users from both samples perceive drug checking as a contribution to risk reduction and that they find providing information for them about the harmful adulterants and substances that they use very important. In addition, users from both samples considered accessibility of the drug checking service as very important and would be in favour of brief counselling at the collection of the drug sample. One of the salient differences between samples was that nightlife drug users found it more important to recognise substances in the drugs that they use.

**Conclusions:**

Drug users from two different samples attach a relatively high importance to the drug checking service, and they consider it to be a contribution to risk reduction. As well as drug users in nightlife settings, high-risk drug users also perceive the drug checking service to be important, which is relevant in the phase of planning drug checking services outside the context of nightlife and for the act of incorporating these services into contemporary harm reduction policy.

## Background

In some countries, drug-checking services for users have been available for more than 15 years, either as a paid or a free service. Since 2001, for example, the Drug Checking service has been available onsite in Zurich [8]. Also, from 2001, a project of the Erowid Centre, called ecstasydata.org, has been active in the USA. Prior to that, from 1999, the organisation DanceSafe provided pill-checking services in the USA, while ChEckiT! from Austria and Energy Control from Spain operated in Europe at that time [5]. In the same period, onsite checking was provided by DROBS (Germany), Energy Control (Spain), and Pilot E (Switzerland) [3]. Despite the numerous positive effects of these programmes [8, 10, 19, 20], such as gaining contact with a hard-to-reach target group and informing about less risky use and emergency interventions [10], there were a relatively small number of onsite checking programmes operating in Europe in 2016 (e.g. Drug Checking, Energy Control, ChEckiT! etc.), where they exist in seven EU countries [2]. The cause of this probably lies in non-harmonised EU legislation or in the restrictions regarding the integration of checking services with drug policy and related legislation [3].

In the early years of drug checking, the development of services coincided with the drug use and nightlife settings of that time. For this reason, the majority of activities were focused on testing MDMA pills or pills sold as MDMA. In the last 10 years, however, there has been a diversification of nightlife in the EU and an ever greater range of psychoactive substances used at parties and in other user environments. According to the EMCDDA reports, recent years have seen a spike in the emergence of new NPSs in Europe [6, 14]. In 2014, 101 new psychoactive substances were discovered by the Early Warning System (EWS) [6, 23]. With the emergence of new substances, drug checking has acquired even further importance in the context of harm reduction. New evaluations and research in this area are crucial for the implementation, broader development, and integration of drug checking services [3, 4, 9]. Given the fact that users who are included in harm reduction programmes in Slovenia [17] and in Hungary [18] inject NPSs (for example 3-MMC), the results of testing and timely warnings on new substances and harmful adulterants may be of great importance for a wider group of users, including those who inject drugs or are not a part of the nightlife environment.

Due to legal restrictions, until now, there has not been any onsite drug checking service that is active in Slovenia. NGO info points for anonymous drug sample collection have been set up since 2006 in two non-governmental organisations (DrogArt and Stigma) as part of the Early Warning System on new psychoactive substances [15]. In 2015 and 2016, as part of the I-SEE^1^ project, the existing EWS system was upgraded with seven new info points for drug sample collection, to then comprise nine info points in total (two for drug users in nightlife settings, six for high-risk drug users, and one for both user groups). After the collection of the sample at the info point, the substance is anonymously confiscated and tested in the National Forensic Laboratory. Afterwards, the result of the analysis (quantitative information is not available to the users) is sent to both the user(s) and the EWS, which then, according to the risk-assessment, proceeds to produce a broader alert. In 2016, two official EWS alerts were issued, based on the results of the anonymously collected samples in Slovenia, which were then circulated to all network members. From these, the warnings were then forwarded to users through NGO channels.

The main purpose of our research was to evaluate the service and the reach of drug checking within the I-SEE project framework in Slovenia and to obtain the views of high-risk drug users, included in harm reduction programmes and drug users in nightlife settings, on drug checking. The high-risk users (mainly users of opioid drugs) were included in the research through seven low-threshold programmes, of which five also provided needle exchange services.^2^ The programmes did not include opioid substitution therapies and were primarily intended for socially excluded users who are injecting drug users and/or whose drug use is perceived as highly risky. Samples were collected in day centres run by these programmes, where users have available space for socialising, psychosocial support and counselling, risk-reduction information, and drug use equipment distribution. The second group, constituted of users in nightlife settings, were included in an online sample which was created with the help of web portals and other channels associated with nightlife.

During the two-year duration of the project, we received 151 samples at the described info points, four of which were NPSs, officially identified for the first time among users in Slovenia (3-MeO-PCP, clonazolam, flubromazolam, and 4-fluoro-butyr-fentanyl). Fifty-six samples included one or more NPSs. Fifteen of these were collected for analysis as a “classic” drug. Apart from the service evaluation, we wished to obtain the users’ views on whether drug checking will encourage drug use, their view of drug checking, and their attitude towards adulterants in the drugs that they use. We were particularly interested in obtaining the opinion of socially excluded high-risk drug users included in harm reduction programmes. An overview of the available literature showed that this is the first research of its kind which addresses the importance of drug-checking services outside the nightlife environment.

## Methods

### Questionnaire design

To assess the situation before designing the questionnaire, we conducted short interviews with employees of organisations involved in the nine harm reduction programmes, where info points were set up. We conducted nine interviews with ten questions related to the drug checking service (e.g. “What was the users’ reaction to the service?”, “What, in your opinion, are the advantages of the drug checking service?”). Based on the answers, provided by professionals working on the programmes, and the possibilities related to conducting the research, our research team designed the questionnaire. In the interpretation phase, we complemented the users’ answers with those from the employees, where and if this was applicable.

We designed a short two-page questionnaire. The estimated time for its completion was 5 min. After demographic data, the first set of questions included questions on the use of drugs in the last month, the use of drugs on an average day/party, and a question on frequency of use. Besides the questions on the prevalence of drug use, we also enquired about risky drug use (sharing drug-use equipment, poly drug use, overdose etc.), followed by questions on drug checking and the main obstacles and advantages of drug checking. Sampling within the harm reduction programmes for high-risk drug users and the online questionnaire for drug users in nightlife settings were carried out in parallel; therefore, some questions differed in both questionnaires. Due to the specifics of the samples, the listed substances in the questions on prevalence partly differed, whereas among the questions on risky drug use, one question differed according to the target group (“I share injecting/sniffing utensils”). In addition, the answers to two specific questions on drug checking differed somewhat according to the target group (“Where did you find out about the drug checking service?”, “What has bothered you so far about the drug checking service?”). For most of the questions, the questionnaires did not differ.

### Sampling

The research took place between November 2016 and the beginning of January 2017. For the purpose of evaluating the drug checking service and obtaining the users’ opinion of the service, which was offered in Slovenia in 2016, we created two different samples. The first sample covered 104 users from seven harm reduction programmes in Slovenia. In this way, we collected 102 completed questionnaires.

The other sample (online questionnaire) covered 610 drug users in nightlife settings. For the purpose of analysis, we excluded partially completed questionnaires to then obtain a final sample of 554 completed questionnaires.

Field sampling (in day centres and as part of field work with the harm reduction programme vans) was carried out by professionals employed in the programmes, who handed out questionnaires to all the users that wished to partake in the research. The collection of questionnaires took place between 16 and 25 December 2016. To ensure a higher level of anonymity, the users included in the harm reduction programmes returned the questionnaires by putting them into closed boxes. Sampling with the online questionnaire took place from mid-December 2016 to the beginning of January 2017. During this time, the questionnaire was publicised on various web portals and forums related to nightlife. The vast majority of respondents accessed the website directly from social media, while a lower number came from web portals and forums. One thousand three hundred fifty-six people clicked on the cover letter, and 746 of these submitted inadequately completed questionnaires. From the remaining 610 questionnaires, we included in the analysis 554 questionnaires from people who also completed the set of questions on the characteristics of drug use since, apart from the data on the evaluation of the drug checking service, we also wanted to obtain those on prevalence and trends in drug use.

## Results

### Sample characteristics

The sample of 102 high-risk drug users from the harm reduction programmes included 71.7% of men and 28.3% of women. The final sample of 554 drug users in nightlife settings (online sample) was composed of 56.2% of men and 43.8% of women.

The age span of the users from programmes ranged from 17 to 58 years (*M* = 35, Mo = 32) and 14 to 60 years for users from the online sample (*M* = 24, Mo = 21).

The majority of the users from programmes have completed secondary education (60.0%) and primary education (34.0%). A smaller number of respondents indicated that they have completed higher or university education (5.0%) and a Master’s or doctoral education (1.0%) (*n* = 100). The majority indicated that they lived in a small city or town (43.4%), followed by a larger city (37.4%), and a village or countryside (19.2%) (*n* = 99). Most respondents declared themselves to be unemployed (85.3%) at the time of the survey, the rest of them were employed (13.7%) or in education (1.1%) (*n* = 95).

In the sample, created online, the majority of respondents indicated that they have completed secondary education (62.9%), followed by those with higher or university education (26.9%). The rest indicated that they have completed primary education (8.3%) or a Master’s or doctoral education (1.8%) (*n* = 553). Nearly half the respondents from the online sample lived in a larger city (49.1%), while the rest of them came from small cities or towns (33.3%), villages or countryside (17.6%) (*n* = 552). Most of the respondents indicated that they were in education (55.9%); the rest of them were employed (36.5%) or unemployed (7.6%) at the time of the survey.

### Characteristics of drug use

Apart from the use of drugs in the last month and on an average day/party, the research aimed to explore the frequency of the use of drugs and some of the characteristics of risky drug use.

Given the specific differences in both samples, which covered users included in harm reduction programmes and the population of drug users in nightlife settings, differences in the characteristics of drug use were expected.

The majority of the users from the programmes indicated that they had used methadone (63.7%), tranquillisers (52.0%), heroin (49.0%), marijuana (47.1%), and cocaine (41.2%) in the last month. A smaller percentage used other stimulant drugs besides cocaine: MDMA (11.8%), amphetamines (8.8%), and 3-MMC (3.9%) (*n* = 102).

The highest percentage of respondents from the online sample used marijuana (83.4%), MDMA (54.2%), cocaine (38.0%), and amphetamines (31.8%) in the last month. Other listed drugs included hallucinogens (11.9%), tranquillisers (8.9%), 3-MMC (6.8%), GHB/GBL (7.5%), methamphetamine (5.6%), and ketamine (5.1%). 2.4% of respondents indicated that they had tried heroin. Other NPSs or substances which were, until recently, considered as NPSs were tried by a relatively small number of respondents. Namely, 2.1% of respondents tried methylone, 1.5% used 4-cmc/3-cmc, and 1.1% tried NBOMe (*n* = 531).

On the last occasion of drug use, the majority of respondents from harm reduction programmes used methadone (49.0%), followed by tranquillisers (43.1%), marijuana (34.3%), heroin (33.3%), and cocaine (21.6%) (*n* = 102).

At an “average” party, the highest percentage of respondents from the online sample used marijuana (63.0%), followed by MDMA (59.3%), amphetamines (32.9%), and cocaine (27.7%). From the list of other drugs, they used 3-MMC (4.9%), GHB/GBL (4.3%), and methamphetamine (3.6%) (*n* = 535).

The highest percentage of respondents from the harm reduction programmes responded to the question on the frequency of drug use by stating that they used drugs on a daily basis (41.0%). A lower percentage (12.0%) stated that they used drugs a few times a week or year. Eleven percent of them used drugs several times per month, but less than once a week. Fourteen percent of respondents from the harm reduction sample stopped using drugs (*n* = 100).

Most of the respondents from the online sample used drugs a few times per year (22.0%) and several times per month, but less than once a week (21.8%). 19.7% of respondents used drugs once a month, 12.3% a few times per week, and 10.0% once a week. The smallest percentage of respondents (6.6%) used drugs on a daily basis. 7.7% of respondents stopped using drugs (*n* = 519).

For the purpose of comparing samples, we excluded respondents who indicated that they had stopped using drugs. Regarding the frequency of drug use, the difference between samples is statistically significant (*T* test; *p* < 0.001). This means that respondents from programmes used drugs significantly more often. Within samples, there are differences regarding the frequency of drug use between genders only in the online sample (*T* test; *p* < 0.05), with men using drugs slightly more often than women (*n* = 478).

We examined the occurrence of *risky drug use* in both samples. The criteria that we established based on our previous research [17] on the use of stimulants and NPS in the context of nightlife included the following: injecting, sharing utensils, simultaneous use of considerable amounts of drugs, and problems due to an overdose. In the online sample, the criterion of injecting was replaced by the criterion of the purchase of drugs immediately before use. A four-level scale was used to examine all the criteria (never, occasionally, often, and always).

Nearly half the respondents from the harm reduction programmes injected drugs, with 21.7% of them reporting doing this often and 22.8% reporting doing it always. A total of 69.6% of respondents injected drugs (*n* = 92). 14.6% of respondents occasionally shared injecting utensils, while 84.1% never did this (*n* = 82). 14.8% indicated that they had simultaneously used considerable amounts of drugs (combined answers of those who responded “often” and “always”) (*n* = 81). 14.8% of respondents (considering those who responded with “occasionally”) already encountered problems related to an overdose (*n* = 81).

45.3% of respondents from the online sample occasionally bought drugs immediately before use (e.g. at a party), 17.9% did this often, while 7.3% reported always doing it (*n* = 501). More than half the respondents indicated sharing sniffing utensils (56.9%). This was occasionally done by 34.8% of them, often by 15.3%, and always by 6.8% (*n* = 503). The simultaneous use of considerable amounts of drugs is characteristic of 48.7% of respondents, out of whom 36.7% do it occasionally, 9.8% often, and 2.2% always (*n* = 501). In the online sample, 20.1% of users had already encountered problems related to an overdose (*n* = 502).

We checked the criterion of the simultaneous use of drugs as a criterion of risky drug use in the context of nightlife by observing the chosen combination of various drugs at an “average” party. For example, cocaine and MDMA were used simultaneously by 113 respondents, which accounts for 20.3% of them (51.3% of men and 48.7% of women). 26.7% (53.4% of men and 46.6% of women) combined amphetamines and MDMA, while amphetamines and cocaine were simultaneously used by 13.1% of users from the online sample (50.7% of men and 49.3% of women).

### Users’ evaluation and opinion on drug checking

The second half of the questionnaire was composed exclusively of questions related to drug checking. A smaller number of questions referred to the evaluation of the service of drug checking as part of the I-SEE project, whereas the rest of the questions were aimed at obtaining the users’ views of drug checking in general.

The main findings related to the evaluation of the drug checking services:

- On completion of the project, 77.5% of users from the programmes and 44.5% of users in nightlife settings (online sample) were *informed* about the drug checking service in 2016 (*n* = 102).
- The majority of those included in the programmes were informed about the service by professionals working in the organisations (52.0%) or professionals involved in fieldwork (12.7%) (*n* = 102).
- In the context of nightlife, the highest percentage of users *were informed about the service* by a friend (39.3%). The rest obtained the information from the Internet (38.4%) or from an onsite promoter (30.6%). 13.5% learnt about the service through the media and 12.2% through flyers (*n* = 229, multiple choice question).
- Nearly a third^3^ of users from the programmes reported that they had *already used* the drug checking service (31.3%, *n* = 99), while the number of nightlife drug users who used the service accounted for 17.5% of them (*n* = 223, 7.0% in respect of the whole sample).
- In the assessment of the *outreach of the service*, we asked them if they received feedback on the results of drug checking. 41.8% (*n* = 91) of respondents from the programmes and 44.6% of drug users in nightlife settings (*n* = 224, 18.1% in respect of the whole sample), confirmed that they had obtained information.
- From all those who used the drug checking service, nine users from the programmes said that they *disliked* the location of the info point, while eight of them found the opening hours of the info point unsuitable, much like the inability to collect samples in the field. Eleven respondents from the online sample indicated as a disadvantage the inability to collect samples in the field, while nine stressed the problem of anonymity with collecting samples in small towns.

The main findings related to users’ opinions on the drug checking service:

- Both groups of drug users (those from the harm reduction programmes and from the online sample) perceive drug checking as a *contribution to risk reduction*. 32.7% (*n* = 101) of respondents from the programmes^4^ and 47.6% (*n* = 437) of users from the online sample strongly agree with this. The combined answers of those who answered with “strongly agree” and “agree” account for 80.2% of respondents who agree from the programmes and 87.6% of respondents who agree from the nightlife users.
- Users from both samples found *informing* about the “hazardous”^5^ substances and adulterants very important. This is very important for 60.4% (*n* = 101) of respondents from the programmes and 66.7% (*n* = 450) of the drug users from the online sample. Combining the answers of those who responded with “important” and “very important”, we see that the activity of informing is important for the majority of respondents from the programmes (95.1%) and from the online sample (96.9%).
- *The accessibility of the drug checking services* is very important for users from the programmes (52.9%, *n* = 102) as well as for the drug users from the online sample (48.1%, *n* = 455). Again, most of the respondents from the first group (89.2%) and from the second group (93.4%) agree with this.
- *Recognition of* “*potentially hazardous*” *substances in drugs that they use* is more important for the drug users from the online sample (56.3%, *n* = 455) than users from the harm reduction programmes (16.3%, *n* = 98). Combining the answers, the majority of respondents from the online sample (95.6%) and more than a third of respondents from the programmes (34.3%) agree with this.
- Users from both groups believe that the drug checking service *does not encourage the use of drugs*. 85.9% (*n* = 99) of respondents from the programmes and 88.4% (*n* = 441) of the nightlife drug users did not agree with this (combined answers of those who responded with “I don’t agree at all” and “I don’t agree”), whereas 14.1% of respondents from the programmes and 11.6% of the drug users from the online sample agreed with the statement.
- The main reasons *for the use* of the drug checking service in both samples are distrust in the quality of substances on the market, risk reduction, and the users’ wish to get information before use of the drug.
- As the principal reasons *discouraging them from using the service*, users from the programmes stated that they use drugs tried by others, that the waiting period for the results is too long, and that they fear the loss of anonymity. Among the drug users from the online sample, the main reasons to avoid the service are the fear of police accessing the data on users, the loss of anonymity, and waiting too long for the results.
- Users from both samples do not mind *brief counselling* at the collection of drug samples (59.4% users from the programmes, *n* = 96 and 58.4% of the users from the online sample, *n* = 457).
- Users from the harm reduction programmes stated that *they would be willing to wait* for the results for up to 2 months (55.0%), while users from the online sample indicated a period of up to 1 week (48.9%). 31.7% (*n* = 454) of respondents from the online sample and 13.0% of respondents from the programmes would be willing to pay for a more rapid test.
- 18.6% (*n* = 97) of respondents from the programmes and 42.7% (*n* = 457) of respondents from the online sample were familiar with quick drug checking tests (e.g. EZ test, Marquis).

Out of six comparisons of the two samples regarding the users’ opinion on the accessibility of drug checking services, the activities of informing users, encouraging drug use, contribution to risk reduction, recognition of used substances, and waiting periods for the results, only the last three topics are characterised by statistically significant differences. Compared to the respondents from the harm reduction programmes, the contribution to risk reduction seems to be of greater importance to the drug users in nightlife settings (95% CI = 0.119–0.457; *T* test; *p* < 0.001), as well as the recognition of potentially hazardous substances in the drugs used by them (95% CI = 1.165–1484; *T*-test; *p* < 0.001). Apart from this, the drug users in nightlife settings are willing to wait less time for the results, compared to users from the programmes (95% CI = −1.682–-1349; T-test; *p* < 0.001).

## Discussion

Our research included two different target groups of drug users, identified as those with a higher prevalence of drug use or higher drug-related risk, compared to the general population of Slovenia [13]. The group of users included in the online survey can be compared to the users from similar research in Zurich [8], in which case the data from our sample on drug use at the “last party/occasion” are similar or slightly higher. The group of injecting drug users, who are included in harm reduction programmes, is considered to be a high-risk group, due to their user habits. The data gathered by our research can be, with certain limitations, compared with the data from research conducted in harm reduction programmes in Slovenia in 2013 and 2014 [11, 12], where use in the last month recorded by our research was lower than the lifetime prevalence in the abovementioned research.

Though our two samples differ with regard to the drugs used, the frequency of drug use and the characteristics of risky drug use, the users for the most part agree with the statements related to drug checking. Data show that most respondents, either those from the programmes (high-risk drug users) or those from the online sample agree that drug checking represents a contribution to risk reduction and that informing users on harmful adulterants is important.

It is possible to link the research findings on the attitude of users from both samples to brief information when the results are delivered, with the results of the first evaluation of drug checking programmes [10], since drug checking enables us to reach hard-to-reach users. For some of them, acquiring information upon results delivery represents the first contact with the harm reduction programme and is not associated merely with the substance, but also with use and with harm reduction. Research carried out by Benschop et al. [1] showed that service users considered counselling, as part of the drug checking service, to be very important. Two research contributions showed that information services, combined with drug checking services, were linked to unaltered drug use [8] or to restricted consumption among MDMA users [1, 8]. The opinion of the users from both samples, according to which they agree that drug checking does not encourage drug use, can be linked to the research findings of the drug checking service evaluation from Zurich where, similarly, results showed that the checking service, combined with the information service, did not encourage drug use [8].

Results showed that the main obstacles to the use of the drug checking system in Slovenia, according to users, were the fear of losing anonymity, long waiting periods for the results, and the unavailability of the checking service in the field. The professionals from the programmes also pointed out legal difficulties related to in-the-field sample collection in vans and the need to obtain quantitative data of the analysis. In the future, we need to improve drug-checking services mainly by ensuring better accessibility of the services, reducing waiting periods for results, informing users about drug-checking services, and developing information and counselling services offered to users upon delivering samples. A drug-checking service may easily be the user’s first contact with harm reduction programmes which may contribute to risk reduction for users in the case that new substances emerge.

Apart from the drug checking services, the EWS also plays a crucial role in the EU. The system currently monitors 560 substances, which accounts for more than half of the substances monitored within the framework of the United Nations Convention [7]. In the light of the emergence of numerous new substances and the results of our research on the importance of informing users on the substances that they use, the EWS could be complemented, based on experience from Slovenia. In this sense, the system would, given that it is the closest system to the integrated warning regime and that it is operating in numerous Member States, collect and transmit not only the information on NPSs, but also information on harmful adulterants present in traditional illicit drugs. The results of the I-SEE project evaluation showed that an increase in the number of NGO info points as part of the EWS in Slovenia contributed to a major number of collected samples and also had a positive impact on informing services for the users. Much like the users, the professionals employed in the harm reduction programmes (with drug checking service) considered the information exchange and information campaigns on NPSs as part of the EWS to be useful and positive. The research was limited to the views and opinions of the users and professionals working in harm reduction programmes. Therefore, to obtain a comprehensive evaluation of the EWS and the effects of drug checking, an evaluation of the effects of drug checking and an evaluation of the effects of the information campaigns on risky use patterns within different user groups should be conducted in the future. This evaluation should also consider other professionals and services which are included in information exchange within EWS (e.g. regional EWS coordinators, representatives of the police, representatives of the Ministry of Health, healthcare professionals etc.).

The research, which has so far emphasised the importance of drug checking in the nightlife context and the integration of such an approach into the concept of harm reduction and into a broader prevention concept [8, 10, 16, 21, 22], as well as within crypto markets [5] can be, based on the results that we obtained, complemented with the importance of drug checking in harm reduction programmes for high-risk drug users.

Ours is probably the first research to have ever revealed the opinion of high-risk drug users on the drug checking service. Professionals working in harm reduction programmes for high-risk drug users included in the research agree that raising the awareness of users on the hazardous adulterants in the drugs they use/inject is of vital importance. Informing injecting and other high-risk drug users about the substances is also important because our results showed that they use drugs tried by others and that recognising substances that they use is important to only one third of these users. Despite the fact that, according to the results obtained, knowing the substances of the drugs is more important to drug users in nightlife settings, this does not mean that we should abandon our efforts to make drug-checking services accessible to injecting drug users or other high-risk users outside the nightlife environment. Due to a demonstrated higher risk with regard to drug use, such information may be of vital importance for both drug users and those employed in harm reduction programmes.

## Conclusions

The results of this research shed a light on the opinion of users from two different groups on the drug checking service as a contribution to harm reduction. The results may furthermore contribute to the development of drug checking services at the local level and may even encourage a reflection on the importance of drug checking services outside the context of nightlife in the EU, since some of the NPSs are now being used (injected) in other user environments as well. The development of a broader drug checking system and rapid delivery of results would enable faster monitoring of changes in the drug market. Combined with accessibility and proper transfer of information, this would reduce the potential harms for users.

## Abbreviations

3-MeO-PCP: 3-Methoxyphencyclidine
3-MMC: 3-Methylmethcathinone
EU: European Union
EWS: Early Warning System
GBL: Gamma-Butyrolactone
GHB: Gamma-hydroxybutyrate
I-SEE: Project for strengthening information exchange between Italy and South-East Europe neighbouring countries on new psychoactive substances

## Substances

MDMA: 3,4-Methylenedioxymethamphetamine
NGO: Non-governmental organisation
NPS: New psychoactive substances

## Footnotes

1 The EU project on new psychoactive substances included five partners: the University of Florence (Italy), the National Institute of Public Health (Slovenia), the Ministry of the Interior (Slovenia), the DrogArt Association (Slovenia), and the School of Medicine from the University of Split (Croatia). https://www.dss.unifi.it/vp-102-i-see.html

2 Hereinafter, we will refer to them as “harm reduction programmes”.

3 Compared to the duplicate data on the collected drug samples in the programmes during the duration of the project, this number should be somewhat lower. This probably means that users interpreted the service as more than merely presenting their sample for checking (e.g. also as obtaining information about the drug checking service in the programme in which they are included).

4 The comparison included a scale from 1 to 4, where 1 was “not important at all” and 4 was “very important”, or “I don’t agree at all” or “I strongly agree”.

5 The questionnaire used the terms “hazardous substances and adulterants” and “potentially hazardous”, since these are used in Slovenia and are clearly understood by users as substances that bring a higher risk of medical complications and overdose.
